# Striatal and peripheral dopaminergic alterations related to cognitive impairment in patients with schizophrenia

**DOI:** 10.1017/S0033291724002228

**Published:** 2024-11

**Authors:** Kai-Chun Yang, Bang-Hung Yang, Chen-Chia Lan, Mu-N Liu, Yuan-Hwa Chou

**Affiliations:** 1 Department of Psychiatry, Taipei Veterans General Hospital, Taipei, Taiwan; 2 Department of Psychiatry, School of Medicine, National Yang Ming Chiao Tung University, Taipei, Taiwan; 3 Department of Nuclear Medicine, Taipei Veterans General Hospital, Taipei, Taiwan; 4 Department of Biomedical Imaging and Radiological Sciences, National Yang Ming Chiao Tung University, Taipei, Taiwan; 5 Department of Psychiatry, Taichung Veterans General Hospital, Taichung, Taiwan; 6 The Human Brain Research Center, Taichung Veterans General Hospital, Taichung, Taiwan

**Keywords:** cognitive impairment, dopamine transporter, phenylalanine, schizophrenia, tyrosine

## Abstract

**Background:**

Cognitive impairment, a major determinant of poor functioning in schizophrenia, had limited responses to existing antipsychotic drugs. The limited efficacy could be due to regional differences in the dysregulation of the dopamine system. This study investigated striatal and peripheral dopaminergic makers in schizophrenia and their relationship with cognitive impairment.

**Methods:**

Thirty-three patients with schizophrenia and 36 age- and sex-matched healthy controls (HC) participated. We evaluated their cognitive performance, examined the availability of striatal dopamine transporter (DAT) using single-photon emission computed tomography with 99mTc-TRODAT, and measured plasma levels of dopaminergic precursors (phenylalanine and tyrosine) and three branched-chain amino acids (BCAA) that compete with precursors for brain uptake via ultra-performance liquid chromatography.

**Results:**

Schizophrenia patients exhibited lower cognitive performance, decreased striatal DAT availability, and reduced levels of phenylalanine, tyrosine, leucine, and isoleucine, and the ratio of phenylalanine plus tyrosine to BCAA. Within the patient group, lower DAT availability in the left caudate nucleus (CN) or putamen was positively associated with attention deficits. Meanwhile, lower tyrosine levels and the ratio of phenylalanine plus tyrosine to BCAA were positively related to executive dysfunction. Among all participants, DAT availability in the right CN or putamen was positively related to memory function, and plasma phenylalanine level was positively associated with executive function.

**Conclusions:**

This study supports the role of dopamine system abnormalities in cognitive impairment in schizophrenia. The distinct associations between different dopaminergic alterations and specific cognitive domain impairments suggest the potential need for multifaceted treatment approaches to target these impairments.

## Introduction

Schizophrenia, a chronic and debilitating psychiatric disorder, causes substantial socioeconomic burdens worldwide (Cowman et al., 2021). Beyond core symptomatology, cognitive impairment has been recognized as the primary impediment to functional recovery in patients, independent of other clinical characteristics (Cowman et al., 2021; Green, Horan, & Lee, 2019; McCutcheon, Keefe, & McGuire, 2023). Despite advances in the development of new antipsychotic drugs, current treatment strategies demonstrate limited efficacy in ameliorating cognitive impairment, leaving many patients struggling to recover (Cowman et al., 2021; McCutcheon et al., 2023; Sinkeviciute et al., 2018). Therefore, to develop more effective treatment strategies, it is crucial to elucidate the neurobiological mechanisms underlying cognitive impairment in schizophrenia.

Dopaminergic dysfunction is one of the leading theories for the pathophysiology of schizophrenia (McCutcheon, Krystal, & Howes, 2020). Dopamine plays a critical role in various cognitive functions across healthy individuals and patients with neuropsychiatric disorders (Cropley, Fujita, Innis, & Nathan, 2006; Takano, 2018). However, current approved antipsychotic drugs, effective in managing psychosis through blockade of dopamine *D*_2_ receptors, show limited success in improving cognitive impairment (Lee et al., 2021; McCutcheon et al., 2023; Sinkeviciute et al., 2018). This disparity could be attributed to regional or circuit-specific dopamine abnormalities that contribute to the distinct clinical characteristics of schizophrenia (Howes, Bukala, & Beck, 2024; Weinstein et al., 2017). For instance, subcortical hyperdopaminergia has been linked to psychosis, while cortical hypodopaminergia is suggested to be associated with cognitive impairment or negative symptoms (Howes et al., 2024; Weinstein et al., 2017). Given the involvement of various mechanisms to maintain dopaminergic homeostasis in different brain regions (Brodnik, Double, España, & Jaskiw, 2017; Howes et al., 2024; Weinstein et al., 2017), an integrated approach is required to concurrently evaluate multiple aspects of the dopamine system to elucidate their roles in cognitive impairment in schizophrenia.

Molecular imaging is the main tool for evaluating the dopamine system in the human brain (Brugger et al., 2020; Cumming, Abi-Dargham, & Gründer, 2021). Striatum and a few subcortical regions were the primary target regions. Low levels of dopaminergic markers in other brain regions made reliable quantification difficult using conventional techniques available in clinical settings (Howes et al., 2024; McCutcheon et al., 2020; Weinstein et al., 2017). Dopamine transporter (DAT) can regulate dopaminergic homeostasis (Vaughan & Foster, 2013). In the human brain, DAT is mainly disturbed in the striatum (Hall et al., 1999), potentially explaining the more prominent role of the norepinephrine transporter in regulating cortical dopamine (Morón, Brockington, Wise, Rocha, & Hope, 2002). Research on striatal DAT availability in schizophrenia has yielded inconsistent findings (Brugger et al., 2020; Howes et al., 2012), and the link to cognitive impairment remains elusive due to a paucity of studies (Yang, Chen, Liu, Yang, & Chou, 2022a). Interestingly, altered DAT levels could be specific to a subgroup of patients due to the high variability in striatal DAT availability in schizophrenia (Brugger et al., 2020). Considering the consistent associations between striatal DAT and cognition in various clinical groups (Chung et al., 2018; Cropley et al., 2006; Yang et al., 2022a), cognitive impairment could be a defining characteristic in patients with altered DAT levels. Therefore, the role of striatal DAT in cognitive impairment in schizophrenia deserves further investigation.

In addition to DAT, maintaining cerebral dopamine homeostasis necessitates a complex interplay between synthesis, reuptake, and release, which are tightly regulated (Best, Nijhout, & Reed, 2009; Okusaga et al., 2014; Yamamoto et al., 2021). Dopamine synthesis requires its precursor amino acids, phenylalanine and tyrosine, and brain levels of these precursors depend on blood concentrations (Bjerkenstedt et al., 2006; Leppik et al., 2018; Wiesel et al., 2005). Moreover, branched-chain amino acids (BCAA), including leucine, isoleucine, and valine, compete with these precursors for the same transport mechanisms across the blood-brain barrier (Bjerkenstedt et al., 2006). Consequently, circulating precursor and BCAA levels can influence precursor uptake and further dopamine synthesis in the human brain. Studies have shown altered blood levels of dopamine precursors (Leppik et al., 2018; Ma et al., 2022; Okamoto et al., 2021; Wei, Xu, Ramchand, & Hemmings, 1995) or BCAA (Leppik et al., 2018; Ma et al., 2022; Okamoto et al., 2021) in schizophrenia, although inconsistent results have also been reported (Davison, O'Gorman, Brennan, & Cotter, 2018; De Luca et al., 2012). Significantly, these alterations have been associated with cognitive impairment (Ma et al., 2022; Wiesel et al., 2005). Furthermore, phenylalanine and tyrosine are known to impact various cognitive domains in different clinical populations (Aquili, 2020; Dora et al., 2023; McCann et al., 2021; Tarn & Roth, 1997). Accordingly, investigating the blood levels of dopamine precursors and BCAA can help elucidate the role of the dopamine system in cognitive impairment in schizophrenia.

While striatal DAT and blood levels of dopamine precursors and BCAA are crucial aspects of the dopamine system that could contribute to cognitive impairment in schizophrenia, no research has yet examined how these dopaminergic markers interact. This study addresses this gap by employing an integrated approach to investigate the roles of striatal DAT and peripheral dopamine precursors/BCAA in cognitive impairment in schizophrenia. We hypothesized that patients with schizophrenia would exhibit poorer cognitive performance, reduced DAT availability, and altered plasma concentrations of dopamine precursors and BCAA compared to healthy controls (HC). Both altered levels of DAT and dopamine precursors would be associated with cognitive impairment.

## Methods

### Participants

This study received approval from the Human Ethics Committee of Taipei Veterans General Hospital (approval numbers: 2016-02-005B and 2017-12-011BC). All participants provided written informed consent before participating, and the research adhered to the Declaration of Helsinki in its latest revision. We recruited patients diagnosed with schizophrenia according to the criteria in the *Diagnostic and Statistical Manual of Mental Disorders, Fifth Edition*, aged between 20 and 65 years. HC were recruited from the community within the same age range and ensured that they had no first-degree relatives with a history of psychiatric disorders. Exclusion criteria were major psychiatric disorders (excluding schizophrenia in patients) or substance use disorders, as identified by the Mini-International Neuropsychiatric Interview, and a history of serious neurological or somatic problems. Participants who received medications significantly affecting DAT imaging were excluded (Chahid, Sheikh, Mitropoulos, & Booij, 2023). We rated the severity of psychopathology in patients using the Positive and Negative Syndrome Scale (PANSS) (Kay, Fiszbein, & Opler, 1987). We converted the doses of their antipsychotic drugs into the chlorpromazine equivalent doses (Leucht, Samara, Heres, & Davis, 2016).

### Cognitive examination

A cognitive battery was administered, comprising tests to assess attention (the Go/No-Go task of Test for Attentional Performance (TAP) and the Color Trails Test), memory (the Word Lists Test and the Face Test of Wechsler Memory Scale-III), and executive function (the Wisconsin Card Sorting Test) in all participants. We computed the composite scores for each cognitive domain based on established methods (Yang, Hsieh, & Chou, 2022b), with the details of the scoring process provided in the online Supplementary Methods.

### Measurement of plasma marker levels

We measured plasma levels of dopaminergic precursors and BCAA using the Acquity ultra-performance liquid chromatography (UPLC) system (Waters, Milford, MA, USA) featuring a fluorescence detector. The online Supplementary Methods provide additional details on sample collection, preprocessing, and analytical methodologies.

### Quantification of striatal DAT availability

Participants underwent SPECT examinations with 99mTc-TRODAT using a two-head gamma camera (E-Cam Variable Angle; Siemens Medical Systems, Erlangen, Germany) equipped with a low-energy fan-beam collimator (Yang et al., 2022a). Magnetic resonance images were acquired using a 3.0 Tesla scanner (MR750, GE Medical Systems, Milwaukee, WI, USA) to exclude underlying intracranial pathology. We used a validated quantitative software optimized for 99mTc-TRODAT (C.-H. Wu, Yang, Chou, Wang, and Chen, 2018) to calculate the specific uptake ratio to indicate DAT availability in four regions of interest: bilateral caudate nucleus (CN) and putamen. The occipital lobe served as a reference region. The online Supplementary Methods reported more details on image acquisition.

### Statistical analysis

We employed R software (version 4.3.1, http://www.R-project.org) for statistical analyses. A two-tailed alpha level of 0.05 was predetermined for statistical significance, maintained before and after multiple comparison adjustments using the Benjamini–Hochberg procedure. The normality of continuous variables was evaluated using the Shapiro–Wilk test. Non-normally distributed variables were transformed using the R package bestNormalize (online Supplementary Table S1).

Demographic differences between groups were examined using independent *t* tests for continuous variables and χ^2^ tests for categorical variables. We used an analysis of covariance (ANCOVA) to assess group differences in cognitive performance and biological markers. Age and sex were covariates in all ANCOVA models. Additionally, years of education served as a covariate in cognitive models, while body mass index (BMI) was incorporated as a covariate for group comparisons of plasma markers.

General linear models (GLMs) were used to explore the associations between biological markers and cognition and the relationship between plasma markers and DAT availability. Age and sex were covariates in all GLMs, with years of education incorporated in models assessing cognition. We implemented a two-step analytical approach to address potential group differences in these relationships (Yang et al., 2021). Initially, interaction terms between the group and the independent variable were incorporated into the initial GLMs to assess whether the relationships varied between the groups. Significant interaction effects prompted separate modified GLMs within each group. Conversely, models without significant interactions used all participants for subsequent modified GLMs. Finally, we calculated effect sizes for the outcomes of independent *t* tests, χ^2^ tests, and ANCOVA/GLMs using Cohen's *d* (suggested interpretation thresholds: small = 0.2, medium = 0.5, large = 0.8), Cramér's *V* (small = 0.1, medium = 0.3, large = 0.5), and partial epsilon-squared (ε2) (small = 0.01, medium = 0.06, large = 0.14) (Cohen, 1992).

## Results

### Participant characteristics

The study included 33 patients with schizophrenia and 36 HC matched for age and sex. Schizophrenia patients exhibited lower education and higher BMI levels compared to HC (Table 1). In terms of clinical characteristics of schizophrenia patients, the duration of the illness was 20.6 ± 6.1 (mean ± standard deviation) years (range: 8–40 years) with an age range of 25–58 years. Patients received antipsychotic drugs with a chlorpromazine equivalent dose of 289.7 ± 189.8 mg per day (see online Supplementary Table S2 for details) and various psychotropic medications, including anxiolytics/hypnotics (*n* = 22), anticholinergics (*n* = 12), antidepressants (*n* = 3), and valproate (*n* = 3). Additionally, seven patients with metabolic problems were prescribed antihypertensive (*n* = 6), antihyperlipidemic (*n* = 3), and antidiabetic (*n* = 1) medications. Their PANSS scores were 53.6 ± 14.8 (total), 12.6 ± 4.7 (positive symptoms), 15.0 ± 7.3 (negative symptoms), and 26.0 ± 7.2 (general psychopathology).

**Table 1.** Comparisons of characteristics between healthy controls and patients with schizophrenia

	HCs	Schizophrenia	*t*	*p*	ES
Sample size	36	33	–	–	–
Age (years)^#^	38.4 ± 10.6	39.6 ± 9.8	−0.54	0.59	−0.13^a^
Sex (female/male)	18/18	16/17	–	1.00^b^	<0.01^c^
Smoking (yes/no)	5/31	4/29	–	1.00^b^	<0.01^c^
Education (years)^#^	14.8 ± 2.5	13.4 ± 2.7	2.31	0.02*	0.56^a^
Body mass index^#^	23.4 ± 3.5	26.3 ± 4.8	−2.62	0.01*	−0.64^a^

ES, effect size; HCs, healthy controls.

Mean ± standard deviation.

a Cohen's *d.*

b χ^2^ test with Yates' continuity correction (df = 1).

c Cramér's *V.*

# Values are normalized before statistical analysis.

* *p* value < 0.05 (independent *t* test, two-tailed).

### Group comparisons of cognition and biological markers

Compared to HC, patients with schizophrenia showed significantly poorer performance in most cognitive subtests (9 out of 13), reflected by lower composite scores across all three cognitive domains: attention (*F*_(1,62)_ = 20.47, *p* < 0.001, partial *ε*^2^ = 0.15), memory (*F*_(1,62)_ = 13.70, *p* < 0.001, partial *ε*^2^ = 0.30), and executive function (*F*_(1,61)_ = 5.54, *p* = 0.022, partial *ε*^2^ = 0.21) (Table 2). Additionally, the patient group exhibited reduced DAT availability in all four striatal regions, decreased plasma levels of two dopaminergic precursors (phenylalanine and tyrosine) and two BCAA (leucine and isoleucine), and a lower ratio of phenylalanine plus tyrosine to BCAA than HC. Importantly, all these observed group differences remained significant after adjusting for multiple comparisons (online Supplementary Table S3). Furthermore, within the schizophrenia group, we found no significant relationships between clinical characteristics and either cognition or biological markers.

**Table 2.** Comparisons of cognitive performance and biological markers between healthy controls and patients with schizophrenia

	HCs (*n* = 36)	Schizophrenia (*n* = 33)	df	*F*	*p*^a^	ES^b^
Go/No-Go task of TAP						
Median of reaction time	441.5 ± 71.7	504.8 ± 91.0	1, 62^c^	7.96	0.006*	0.10
Color Trails Test						
Trails 1 time^#^	36.6 ± 12.9	51.5 ± 20.3	1, 62^c^	9.35	0.003*	0.12
Trails 2 time^#^	70.9 ± 19.6	117.9 ± 61.5	1, 62^c^	17.42	<0.001*	0.21
Word lists test of WMS-III						
Immediate: Word lists I recalls	37.5 ± 4.0	31.2 ± 8.5	1, 62^c^	11.19	0.001*	0.14
Delayed: Word lists II recalls^#^	9.9 ± 1.4	8.1 ± 2.7	1, 62^c^	8.20	0.006*	0.10
Retention (%)^#^	90.0 ± 9.6	81.5 ± 17.8	1, 62^c^	1.95	0.168	0.01
Face memory test of WMS-III						
Immediate: Face recognition I	38.0 ± 4.4	34.7 ± 4.2	1, 62^c^	8.16	0.006*	0.10
Wisconsin Card Sorting Test						
Errors (%)^#^	22.7 ± 14.8	33.4 ± 17.1	1, 61^d^	6.58	0.013*	0.08
Perseverative responses (%)^#^	11.3 ± 7.9	21.6 ± 18.4	1, 61^d^	8.02	0.006*	0.10
Perseverative errors (%)^#^	10.6 ± 6.8	18.5 ± 13.9	1, 61^d^	7.06	0.010*	0.09
Nonperseverative errors (%)^#^	12.0 ± 9.3	15.1 ± 9.2	1, 61^d^	1.81	0.183	0.01
Conceptual level responses (%)^#^	69.6 ± 23.3	57.0 ± 23.8	1, 61^d^	3.30	0.074	0.04
Number of categories completed^#^	5.3 ± 1.6	4.5 ± 2.1	1, 61^d^	0.94	0.336	<0.01
Plasma levels of amino acids (*μ*M)						
Phenylalanine	35.8 ± 11.6	24.8 ± 12.6	1, 64	13.78	<0.001*	0.16
Tyrosine^#^	48.2 ± 22.2	37.1 ± 19.2	1, 64	9.84	0.003*	0.12
Leucine	92.2 ± 31.1	74.6 ± 24.6	1, 64	10.54	0.002*	0.13
Isoleucine	57.4 ± 21.5	46.2 ± 16.5	1, 64	9.24	0.003*	0.11
Valine^#^	209.7 ± 45.2	211.3 ± 56.8	1, 64	0.35	0.559	<0.01
Phe + Tyr/BCAA ratio^#^	0.23 ± 0.07	0.19 ± 0.09	1, 64	5.23	0.026*	0.13
Availability of dopamine transporter						
Left CN^#^	1.77 ± 0.30	1.53 ± 0.26	1, 65	13.86	<0.001*	0.16
Right CN	1.71 ± 0.30	1.52 ± 0.32	1, 65	6.42	0.014*	0.08
Left putamen	1.89 ± 0.33	1.62 ± 0.29	1, 65	16.33	<0.001*	0.17
Right putamen	1.81 ± 0.31	1.61 ± 0.32	1, 65	7.08	0.010*	0.08

BCAA, branched-chain amino acid (leucine + isoleucine + valine); CN, caudate nucleus; df, degree of freedom; ES, effect size; HCs, healthy controls; Phe, phenylalanine; TAP, Test for Attentional Performance; Tyr, Tyrosine; WMS-III, Wechsler Memory Scale-III.

Mean ± standard deviation.

a Analyses of covariance with covariates: age, sex, and education (cognitive tests); age, sex, and body mass index (plasma amino acids); age and sex (imaging data).

b Partial epsilon-squared (*ε*^2^).

c There are no data available for two patients with schizophrenia.

d There are no data available for three patients with schizophrenia.

# Values are normalized before statistical analysis.

* *p* value < 0.05 (two-tailed).

### Relationships between cognition and biological markers

Among the cognition and biological markers exhibiting alterations in schizophrenia, there were significant differences between the two groups in the relationships of DAT availability in left CN or putamen with attention scores and of plasma tyrosine level or the ratio of phenylalanine plus tyrosine to BCAA with executive function scores. Consequently, we examined these four pairs of relationships between biological markers and cognition within each group while evaluating other pairs of such relationships across all participants.

**Figure 1.**
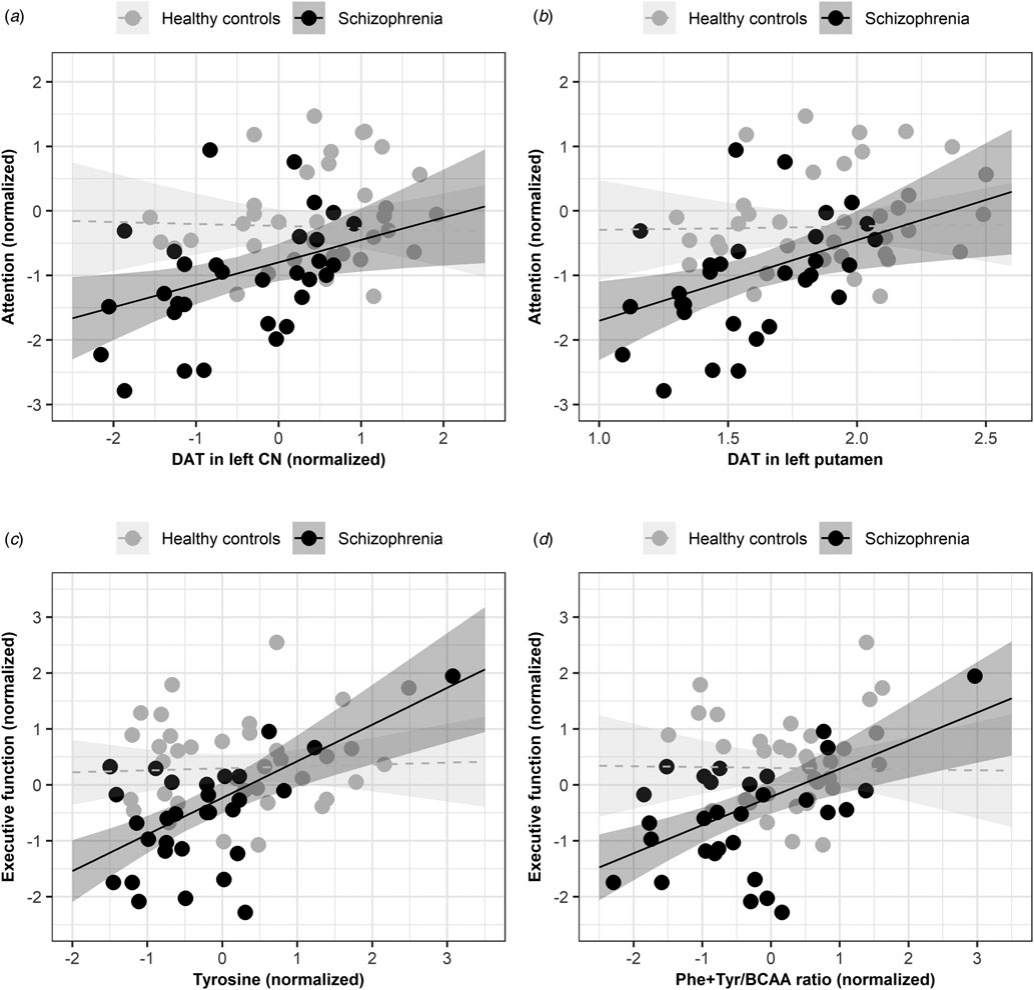
Scatter plots with linear regression lines and 95% confidence intervals illustrate the relationships between biological markers and cognitive performance in healthy controls and patients with schizophrenia. The solid lines indicated significant relationships, while the dashed lines represented nonsignificant ones. Age, sex, years of education, and group membership were covariates. All four models revealed significant group interactions for the relationships. Specifically, significant positive relationships were only observed in patients with schizophrenia but not in healthy controls (a) DAT in left CN and attention. (b) DAT in left putamen and attention. (c) Plasma concentrations of tyrosine and executive function. (d) The ratio of phenylalanine plus tyrosine to BCAA and executive function. BCAA, branched-chain amino acids; CN, caudate nucleus; DAT, dopamine transporter availability; Phe, phenylalanine; Tyr, tyrosine.

For patients with schizophrenia, we observed significant positive relationships between reduced DAT availability in left CN or putamen and attention deficits. Similarly, decreased tyrosine levels or a lower ratio of phenylalanine plus tyrosine to BCAA were associated with worse executive function. HC did not exhibit these relationships (Table 3 and Fig. 1). We then examined all participants and found positive relationships between DAT availability in the right CN or putamen and memory scores. At the same time, plasma phenylalanine levels were positively associated with executive function scores (Table 4). Except for associations between DAT availability and memory scores, all observed relationships retained significance after adjusting for multiple comparisons (online Supplementary Table S4). Furthermore, we did not find significant associations between peripheral markers and striatal DAT availability.

**Table 3.** General linear models for the biological markers associated with cognition in patients with schizophrenia

Dependent variable^a^	Exploratory variable	Standardized *β*	*β*	95% CI low	95% CI high	*t*	*p*	ES^b^
Attention	Left CN DAT^#^	0.345	0.332	0.038	0.627	2.32	0.029*	0.14
	Age^#^	−0.222	−0.212	−0.510	0.086	−1.46	0.156	0.04
	Education^#^	0.497	0.480	0.184	0.776	3.33	0.003*	0.27
	Sex	0.221	0.385	−0.186	0.955	1.38	0.178	0.03
Attention	Left putamen DAT	0.404	1.266	0.319	2.213	2.75	0.011*	0.20
	Age^#^	−0.204	−0.195	−0.484	0.094	−1.39	0.177	0.03
	Education^#^	0.523	0.505	0.219	0.791	3.63	0.001*	0.31
	Sex	0.194	0.338	−0.218	0.895	1.25	0.222	0.02
Executive function	Tyrosine^#^	0.710	0.688	0.414	1.006	4.95	<0.001*	0.47
	Age^#^	−0.315	−0.288	−0.635	0.005	−2.03	0.054	0.11
	Education^#^	0.491	0.467	0.188	0.794	3.34	0.003*	0.28
	Sex	0.208	0.110	−0.348	0.764	0.77	0.449	<0.01
Executive function	Phe + Tyr/BCAA ratio^#^	0.586	0.680	0.313	0.858	4.43	<0.001*	0.42
	Age^#^	−0.397	−0.362	−0.746	−0.047	−2.34	0.028*	0.15
	Education^#^	0.517	0.492	0.195	0.839	3.31	0.003*	0.28
	Sex	0.133	0.070	−0.456	0.721	0.46	0.646	<0.01

BCAA, branched-chain amino acid; CI, confidence interval; CN, caudate nucleus; DAT, dopamine transporter availability; ES, effect size; Phe, phenylalanine; Tyr, Tyrosine.

a Composite score of the cognitive domain.

b Partial epsilon-squared (*ε*^2^).

# Values are normalized before statistical analysis.

* *p* value < 0.05 (two-tailed).

**Table 4.** General linear models for the biological markers associated with cognition in all study participants

Dependent variable^a^	Exploratory variable	Standardized *β*	*β*	95% CI low	95% CI high	*t*	*p*	ES^b^
Memory	Right CN DAT	0.217	0.607	0.136	1.984	2.29	0.025*	0.06
	Age^#^	−0.231	−0.229	−0.323	0.294	−0.09	0.926	<0.01
	Education^#^	0.236	0.253	−0.076	0.488	1.46	0.149	0.02
	Sex	0.129	0.257	−0.748	0.345	−0.74	0.464	<0.01
	Group	−0.316	−0.621	−1.339	−0.239	−2.87	0.006*	0.10
Memory	Right putamen DAT	0.258	0.917	0.012	1.822	2.03	0.047*	0.05
	Age^#^	−0.036	−0.043	−0.349	0.263	−0.28	0.780	<0.01
	Education^#^	0.169	0.213	−0.073	0.499	1.49	0.141	0.02
	Sex	−0.097	−0.228	−0.783	0.326	−0.82	0.414	<0.01
	Group	−0.337	−0.793	−1.353	−0.232	−2.83	0.006*	0.10
Executive function	Phenylalanine	0.312	0.024	0.007	0.040	2.89	0.005*	0.11
	Age^#^	0.008	0.009	−0.210	0.227	0.08	0.937	<0.01
	Education^#^	0.412	0.438	0.224	0.652	4.10	<0.001*	0.21
	Sex	−0.017	−0.034	−0.447	0.379	−0.16	0.870	<0.01
	Group	−0.225	−0.448	−0.894	−0.002	−2.01	0.049*	0.05

CI, confidence interval; CN, caudate nucleus; DAT, dopamine transporter availability; ES, effect size.

a Composite score of the cognitive domain.

b Partial epsilon-squared (*ε*^2^).

# Values are normalized before statistical analysis.

* *p* value < 0.05 (two-tailed).

## Discussion

This study investigated alterations in striatal DAT and peripheral dopaminergic markers, along with how these abnormalities relate to cognitive impairment in schizophrenia. Compared to HC, patients with schizophrenia exhibited poorer cognitive performance, reduced striatal DAT availability, and lower plasma levels of phenylalanine, tyrosine, leucine, and isoleucine, and the ratio of phenylalanine plus tyrosine to BCAA. Within the schizophrenia group, lower DAT availability in the left CN or putamen was positively associated with attention deficits. Additionally, decreased tyrosine levels and the ratio of phenylalanine plus tyrosine to BCAA exhibited positive associations with executive dysfunction. Furthermore, across all participants, DAT availability in the right CN or putamen showed a positive correlation with memory function, while plasma phenylalanine level was positively associated with executive function.

This work represents the first to combine evaluations of central and peripheral dopaminergic markers to assess their associations with cognitive impairment in schizophrenia. We observed reduced striatal DAT availability related to attention or memory deficits. At the same time, decreased levels of tyrosine, phenylalanine, and the ratio of phenylalanine plus tyrosine to BCAA were correlated with executive dysfunction. These observations expand upon previous research demonstrating links between altered striatal dopaminergic markers and cognitive impairment in schizophrenia (Takano, 2018) and the positive relationships between reduced striatal DAT availability and attention or memory deficits in our previous work (Yang et al., 2022a). Additionally, the present findings align with the associations of tyrosine or phenylalanine with prefrontal cortex-dependent executive functions (Ahrens, Laux, Müller, & Thiel, 2020; Aquili, 2020; Dora et al., 2023).

Interestingly, previous research also indicated that tyrosine supplements primarily increase dopamine levels in the prefrontal cortex compared to the striatum (Brodnik et al., 2017). This regional disparity aligns with the concept of distinct regulatory mechanisms for dopamine homeostasis across brain regions (Howes et al., 2024; Morón et al., 2002; Weinstein et al., 2017). Therefore, although more validation is required, these findings highlight that striatal DAT availability and peripheral dopaminergic markers could be related to distinct cognitive domains. This observation further supports the notion that regionally specific dopamine regulation abnormalities contribute to various cognitive domain impairments in schizophrenia.

The observed reductions in striatal DAT availability in schizophrenia, which correlated with attention or memory deficits, are consistent with the literature (Mateos et al., 2007; Yang et al., 2022a). As the measured availability can indicate the capacity to regulate dopamine or dopaminergic neuron integrity, reduced DAT availability reflects either downregulation of DAT expression or loss of dopaminergic neurons (Smith et al., 2018; Tseng et al., 2017; Vaughan & Foster, 2013). Given the established striatal hyperdopaminergic state in schizophrenia (Howes et al., 2024; Sekiguchi, Pavey, & Dean, 2019) and the associations between striatal DAT availability and attention or memory functions observed across various clinical populations (Cropley et al., 2006; Itagaki et al., 2024; Rieckmann, Johnson, Sperling, Buckner, & Hedden, 2018; Smith et al., 2018), we posit that downregulation secondary to hyperdopaminergia is the primary cause of reduced striatal DAT availability.

Furthermore, the lack of significant alterations in striatal DAT availability in previous meta-analyses (Brugger et al., 2020; Howes et al., 2012) could be attributed to the high variability in DAT availability reported in schizophrenia (Brugger et al., 2020), with reductions potentially limited to a subgroup of patients with significant cognitive impairment (Lewandowski et al., 2019). The clinical characteristics of our schizophrenia patients further support this notion, as most of them exhibited cognitive impairment along with reduced DAT availability, even with good clinical responses evidenced by PANSS scores (White et al., 2016). In summary, our findings solidify the role of striatal DAT in cognitive impairment, particularly attention or memory deficits, in schizophrenia. It is warranted to investigate its implications for managing these cognitive deficits.

Our findings of reduced plasma concentrations of dopaminergic precursors and BCAA in schizophrenia patients corroborate previous studies (Leppik et al., 2018; Ma et al., 2022; Okamoto et al., 2021; Wei et al., 1995), although inconsistent findings were reported (Davison et al., 2018; Okusaga et al., 2014). These discrepancies may arise from the heterogenicity of the recruited schizophrenia patients or the methodologies for quantifying blood concentrations (Davison et al., 2018). Interestingly, the substantial heterogeneity in the studies with results comparable to the present work implied that these observations were not sensitive to methodological heterogeneity. Significantly, despite the observed decrease in plasma BCAA concentrations in schizophrenia, the concomitant reduction in the ratio of phenylalanine plus tyrosine to BCAA further strengthens the notion of diminished dopaminergic precursor availability in these patients.

Moreover, this work is the first to elucidate the cognitive implications of altered plasma dopaminergic precursor concentrations in schizophrenia, demonstrating positive relationships with executive dysfunction. These observations are consistent with reports of decreased blood levels of dopaminergic precursors or BCAAs in patients with dementia or cognitive decline (Conde et al., 2024; Lista et al., 2023) and positive associations between these markers and cognitive function (Aquili, 2020; McCann et al., 2021). As dopamine concentrations in the prefrontal cortex were sensitive to the availability of precursors (Brodnik et al., 2017), the reduced plasma precursor concentrations implicated the prefrontal hypodopaminergic state in these patients. These alterations have been well-documented in schizophrenia and were associated with executive dysfunction (Howes et al., 2024; Weinstein et al., 2017). In summary, the present work supported the roles of decreased plasma levels of dopaminergic precursors in executive dysfunctions in schizophrenia. More research is warranted to explore the potential treatment implications of this association.

The current findings suggest the need for various treatment approaches tailored to the specific cognitive domains of schizophrenia. Tyrosine supplementation has shown promise in enhancing cognitive function, particularly executive function, in different clinical populations (Ahrens et al., 2020; Aquili, 2020; Dora et al., 2023; Thomas, Lockwood, Singh, & Deuster, 1999). However, its application in schizophrenia requires careful consideration due to the reported inverted U-shaped relationship between dopaminergic markers and cognition (Aquili, 2020; Smucny, Dienel, Lewis, & Carter, 2022) and the wide range of dosing regimens employed in prior research (Ahrens et al., 2020; Aquili, 2020). Therefore, selecting an optimal dose is critical for maximizing therapeutic benefit. Additionally, potential adverse effects from elevated striatal dopamine levels following tyrosine supplementation warrant investigation (Howes et al., 2024).

Neuromodulatory techniques such as transcranial direct current stimulation (tDCS) or repetitive transcranial magnetic stimulation (rTMS) offer a promising avenue for regionally selective enhancement of the dopaminergic state (Howes et al., 2024; Smucny et al., 2022). Given the high density of dopamine *D*_1_ receptors in the prefrontal cortex, *D*_1_ receptor agonism presents a logical alternative approach, but relevant medications are not currently available (Smucny et al., 2022). On the other hand, N-acetyl-L-cysteine, an antioxidant shown to enhance DAT expression (Monti et al., 2019), has been reported to improve cognition in patients with schizophrenia (Bradlow, Berk, Kalivas, Back, & Kanaan, 2022). To optimize treatment efficacy, we suggested applying pre-treatment evaluation of relevant dopaminergic markers and impaired cognitive domains to guide therapeutic selection (Conus et al., 2018). Ultimately, these observations can inform future mechanistic and clinical studies that aim to refine treatment strategies for cognitive impairment in schizophrenia.

The current study did not identify a significant relationship between striatal DAT availability and plasma concentrations of dopaminergic precursors. There is also a lack of existing literature on this specific association. On the contrary, prior research has demonstrated relationships between striatal DAT availability and striatal dopamine synthesis capacity (Yamamoto et al., 2021) or blood levels of some amino acids (Hartstra et al., 2020; Yang et al., 2022a), suggesting a possible relationship between striatal DAT availability and blood concentrations of dopaminergic precursors. In light of the established various regulatory mechanisms across brain regions (Howes et al., 2024; Morón et al., 2002; Weinstein et al., 2017), our findings support the hypothesis that the availability of dopaminergic precursors does not primarily regulate striatal DAT availability in schizophrenia. On the other hand, associations between striatal DAT and plasma levels of metabolites involved in the methionine cycle have been reported in schizophrenia (Yang et al., 2022a) and metabolic syndrome (Hartstra et al., 2020), implicating the potential roles of methylation reactions or oxidative stress in DAT expressions (Kim & Andreazza, 2012; Wiers et al., 2018; Yang et al., 2022a). Therefore, the absence of a link between striatal DAT and plasma dopaminergic precursors reinforces the varied regulation mechanisms for different dopaminergic markers, and diverse approaches might be needed to regulate them.

One fundamental limitation of this study is the possible confounding effect of psychotropic drugs administered to schizophrenia patients. Notably, we did not observe significant relationships between antipsychotic drug doses and cognition or biological markers. Despite a recent systemic review (Chahid et al., 2023) reporting that only Haloperidol and Bupropion, neither of which was administered in this study, significantly reduce DAT imaging, the potential influence of other psychotropic drugs on DAT imaging cannot be entirely ruled out. Antipsychotic drugs can have various effects on blood amino acid concentrations (Leppik et al., 2018; Parksepp et al., 2020). These effects include elevations in tyrosine levels (Leppik et al., 2018), contradicting our observations of reduced tyrosine levels in schizophrenia. Moreover, the complex interplay between antipsychotic drugs and cognitive performance, with different medications exerting varying effects (enhancing, nonsignificant, or impairing) on various cognitive domains (Baldez et al., 2021; Lee et al., 2021; Vyas et al., 2018), highlights the significant confounding influence of psychotropic drugs exposure in this study. Future research focused on drug-naive patients is warranted to replicate our findings.

Another limitation is the higher BMI levels observed in patients with schizophrenia. BMI, a known risk factor for metabolic syndrome, can influence both striatal DAT availability and blood amino acid concentrations. A recent review revealed inconsistent findings regarding the association between DAT availability and BMI: one study reported a significant negative correlation (Chen et al., 2008), while four others found no such association (Pak, Seok, Lee, Kim, & Kim, 2023). Conversely, higher BMI or related indices have been linked to increased blood concentrations of dopaminergic precursors and BCAA (Hamaya et al., 2022; Mikkola, Salonen, Kajantie, Kautiainen, & Eriksson, 2020), which contradicts the decreased levels of these markers observed in our patients with schizophrenia. To mitigate the potential confounding effects of BMI differences between groups, we included BMI as a covariate in the group comparison analyses, following the established practice of previous studies reporting higher BMI in patients with schizophrenia (Leppik et al., 2018; Ma et al., 2022; Okusaga et al., 2014).

Similarly, smoking, a prevalent comorbidity in schizophrenia, has a complex interaction with monoamine neurotransmission, potentially influencing striatal DAT availability and blood amino acid concentrations. While research suggests that smoking may reduce tyrosine levels, its impact on striatal DAT availability, phenylalanine, or BCAA concentrations remains inconclusive (Hamaya et al., 2022; Mathai et al., 2016; Pak et al., 2023). Given the comparable smoking rates between our study groups, it is unlikely that observed group differences in biological markers are primarily attributable to smoking. Nonetheless, future studies should apply a more detailed evaluation of smoking-related clinical characteristics to elucidate potential effects.

Furthermore, given that phenylalanine and three BCAA are essential amino acids (National Research Council, 1989), dietary habits can influence our findings, especially the frequent unhealthy nutritional patterns in schizophrenia patients (Wu, Wang, Bai, Huang, & Lee, 2007). Remarkably, comparable reductions in these markers have been observed in diverse clinical populations with established functional implications (Aquili, 2020; Conde et al., 2024; Lista et al., 2023; McCann et al., 2021). These findings suggest that the present changes in schizophrenia were less likely only incidental findings caused by diet.

In addition to previously identified potential confounders, substantial heterogeneity among patients regarding age, illness duration, and clinical stage likely influenced striatal DAT availability and peripheral dopamine precursor levels (Baldez et al., 2021; Brugger et al., 2020). This heterogeneity probably introduced prominent variance in our outcome measures, hindering our ability to detect statistically significant results, particularly for biological markers with inherently higher variability than cognitive measures (Brugger et al., 2020; McCutcheon et al., 2023; Parksepp et al., 2020). Furthermore, while typical in molecular imaging studies, the relatively small sample size limited statistical power and the ability to detect potential non-linear relationships between biological markers and cognition, which precluded a comprehensive investigation of complex interactions among these variables (Aquili, 2020; Takano, 2018). Further methodological limitations included the lack of specific cognitive model validation for schizophrenia patients and the inability to infer causality from the cross-sectional design. Future research should prioritize longitudinal studies to establish causality, employ larger and more homogenous patient groups, utilize validated research instruments, robustly control confounders, and explore potential non-linear relationships.

In conclusion, the present study provided further support for the involvement of the dysregulated dopamine system in cognitive impairment in schizophrenia. The observed decreased striatal DAT availability and peripheral dopaminergic precursors were consistent with the established literature on regional variations in dopaminergic abnormalities in schizophrenia. The distinct associations of striatal and peripheral dopaminergic markers with specific cognitive domain impairments align with the diverse mechanisms of dopamine regulation across brain regions and their functional roles. Future research is warranted to replicate these findings and develop targeted treatment strategies to address specific cognitive domain impairments in schizophrenia.

## Supporting information

Yang et al. supplementary material Yang et al. supplementary material

## Supplementary material

The supplementary material for this article can be found at https://doi.org/10.1017/S0033291724002228
