# Supplementary material for: Striatal and peripheral dopaminergic alterations related to cognitive impairment in patients with schizophrenia
Source: Psychol Med. 2024 Dec 2;54(15):4182–92. doi: 10.1017/S0033291724002228 (PMC11650164; doi:10.1017/S0033291724002228)
Supplement: Yang et al. supplementary material [file S0033291724002228sup001.docx]

**Supplementary Material**

**Supplementary Methods**

**Cognitive examination**

Participants underwent a battery of cognitive subtests that target specific domains. These included the Go/No-Go task of Test for Attentional Performance (TAP) and the Color Trails Test for attention (three subtests). Memory was assessed using the Word Lists Test and the Face Test of Wechsler Memory Scale-III (four subtests). Executive function was evaluated with the Wisconsin Card Sorting Test (six subtests). Our previous work informed us of the selection of these measures and the targeted cognitive domains (attention, memory, and executive functions) (Yang, Hsieh, & Chou, 2022). The raw scores of the cognitive subtests were converted to z-scores using the mean and standard deviation of a healthy control group. To ensure that higher scores reflect better performance on all measures, the generated z-scores were multiplied by -1 as required. Finally, a composite score was calculated for each cognitive domain by averaging the z-scores of the constituent cognitive subtests.

**Measurement of plasma marker levels**

Following catheter insertion, participants remained supine for 15 minutes before blood collection. Venous blood samples were obtained using Vacutainer tubes before radioligand injection. Plasma was isolated via centrifugation at 3000 rpm for 30 minutes at 4°C. Following careful collection, samples were snap-frozen on dry ice and stored at -80°C until analysis. Plasma amino acid concentrations were determined using

an Acquity ultra-performance liquid chromatography (UPLC) system (Waters, Milford, MA, USA) equipped with a fluorescence detector. The analysis adhered to the manufacturer's protocol, employing pre-column derivatization with the AccQ·Tag Ultra Derivatization Kit (Waters, Milford, MA, USA). Briefly, 70 μl of borate buffer was mixed with 10 μl of the sample, followed by the addition of 20 μl of reagent solution. The reaction mixture was immediately vortexed, incubated for 1 minute, and then heated to 55 °C for 10 minutes. The derivatized sample was subsequently injected into the UPLC system. The UPLC analysis utilized an AccQ·Tag Ultra Column (100 mm × 2.1 mm, 1.7 μm column) (Waters, Milford, MA, USA) maintained at a constant temperature of 55°C. An injection volume of 1 μl was employed with a flow rate of 0.7 ml/min. The excitation and emission detection wavelengths were set at 266 nm and 473 nm, respectively. A binary solvent system was utilized, consisting of (A) AccQ⋅Tag-ultra eluent A concentrate (5%, v/v) and water (95%, v/v) and (B) AccQ⋅Tag-ultra eluent B. The elution gradient program was as follows: 0–0.54 min, 99.9% A–0.1% B; 5.74 min, 90.9% A–9.1% B; 7.74 min, 78.8% A–21.2% B; 8.04 min, 40.4% A–59.6% B; 8.05–8.64 min, 10% A–90% B; 8.73–10 min, 99.9% A–0.1% B. Data acquisition, calibration, and quantification were performed using Empower 3 software (Waters).

**Imaging acquisition**

SPECT image acquisition commenced 240-280 minutes post-administration of a single bolus injection of 740 MBq (20 mCi) 99mTc-TRODAT. A two-head gamma camera system (E-Cam Variable Angle; Siemens Medical Systems, Erlangen, Germany) equipped with a low-energy fan-beam collimator was employed for this purpose. The system offered a resolution of 7.3 mm, featuring a pixel size of 3.9 mm and an image matrix of 128 × 128.

The T1-weighted anatomical images were acquired on a 3.0 Tesla scanner (MR750, GE Medical Systems, Milwaukee, WI, USA) with an 8-channel high-resolution brain coil. The parameters of the imaging sequence included repetition time/echo time = 9.184/3.68 ms; field of view = 256 mm × 256 mm; matrix = 256 × 256 × 168; number of excitations = 1; inversion time = 450 ms; flip angle = 12 ° and voxel size = 1 × 1 × 1 mm.

**Supplementary Results**

**
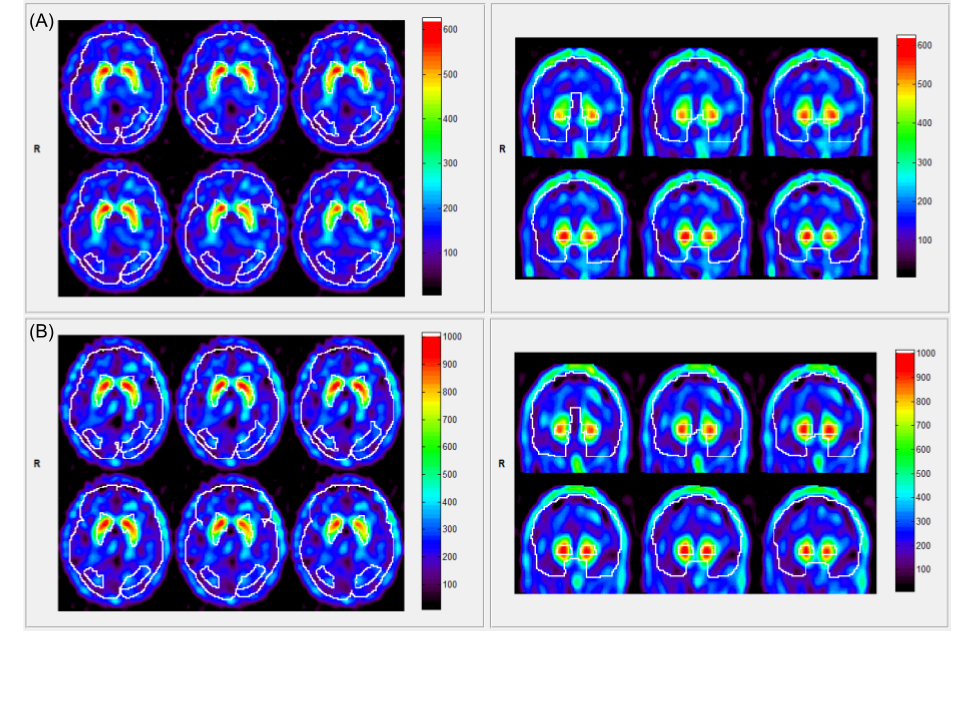
**

**Supplementary Figure 1.** Representative 99mTc-TRODAT images and corresponding regions of interest defined in image analysis software. (A) A healthy control exhibited dopamine transporter (DAT) availability of 1.71 in the left caudate nucleus (CN), 1.76 in the right CN, 1.85 in the left putamen, and 1.81 in the right putamen. (B) Patients with schizophrenia demonstrated DAT availability of 1.59 in the left CN, 1.53 in the right CN, 1.73 in the left putamen, and 1.67 in the right putamen.

**Supplementary Table S1.** Normalizing transformations for nonnormally distributed variables

| Transformations | Variables |
| --- | --- |
| The standardized Asinh transformation | PANSS negative subscale scores  Chlorpromazine equivalent doses  Trails 1 time of Color Trails Test  Nonperseverative errors (%) of WCST |
| The standardized Box Cox transformation | Body mass index  PANSS GP subscale scores  Word lists II recalls of WMS-III  Trails 2 time of Color Trails Test  Perseverative errors (%) of WCST |
| The center and scale transformation | Tyrosine |
| The ordered quantile transformation | Years of education  Duration of illness  Errors (%) of WCST |
| The standardized Log_b(x + a) transformation | Phe+Tyr/BCAA ratio  DAT availability in left CN |
| The standardized double reversed Log_b(x + a) transformation | PANSS positive subscale scores  Word retention (%) of WMS-III  CLR (%) of WCST |
| The standardized square root (x + a) transformation | Age  Completed categories of WCST  Valine |
| The standardized Yeo-Johnson transformation | Perseverative responses (%) of WCST |

BCAA = branched-chain amino acid, CLR = conceptual level responses, CN = caudate nucleus, DAT = dopamine transporter, GP = general psychopathology, PANSS = Positive and Negative Syndrome Scale, Phe = phenylalanine, Tyr = Tyrosine, WCST = Wisconsin Card Sorting Test, WMS-III = Wechsler Memory Scale-III

**Supplementary Table S2.** Doses of antipsychotic drugs in patients with schizophrenia

| Patients | Doses (mg per day) | Chlorpromazine equivalent doses |
| --- | --- | --- |
| 1 | Amisulpride 200 | 150 |
| 2 | Amisulpride 200 | 150 |
| 3 | Ziprasidone 120 | 450 |
| 4 | Aripiprazole 20 | 400 |
| 5 | Aripiprazole 10 | 200 |
| 6 | Risperidone 2 | 120 |
| 7 | Amisulpride 200 | 150 |
| 8 | Amisulpride 400 | 300 |
| 9 | Amisulpride 300 | 225 |
| 10 | Risperidone 4 | 240 |
| 11 | Olanzapine 22.5 | 675 |
| 12 | Aripiprazole 10 | 200 |
| 13 | Aripiprazole 5 | 100 |
| 14 | Ziprasidone 80 | 300 |
| 15 | Aripiprazole 20 | 400 |
| 16 | Aripiprazole 10 | 200 |
| 17 | Risperidone 4 | 240 |
| 18 | Aripiprazole 12.5 | 250 |
| 19 | Quetiapine 650 | 487.5 |
| 20 | Aripiprazole 10 | 200 |
| 21 | Amisulpride 700 | 525 |
| 22 | Aripiprazole 10 | 200 |
| 23 | Amisulpride 400 | 300 |
| 24 | Aripiprazole 10 | 200 |
| 25 | Risperidone 6 | 360 |
| 26 | Paliperidone 9 | 450 |
| 27 | Amisulpride 50 | 37.5 |
| 28 | Aripiprazole 10 | 200 |
| 29 | Amisulpride 800 + Chlorpromazine 400 | 1000 |
| 30 | Amisulpride 200 | 150 |
| 31 | Aripiprazole 20 | 400 |
| 32 | Amisulpride 200 | 150 |
| 33 | Amisulpride 200 | 150 |

**Supplementary Table S3.** The *p* values of group comparison results before and after adjusting multiple comparisons

|  | *p* values (original) | *p* values (adjusted) |
| --- | --- | --- |
| Cognitive domains | | |
| Attention^a^ | 0.00002794^*^ | 0.00008382^*^ |
| Memory^a^ | 0.0004584^*^ | 0.0006876^*^ |
| Executive function^a^ | 0.02181^*^ | 0.02181^*^ |
| Striatal DAT availability | | |
| Left CN^b^ | 0.0004143* | 0.0008286^*^ |
| Right CN^b^ | 0.0137^*^ | 0.0137^*^ |
| Left putamen^b^ | 0.0001433^*^ | 0.0005732^*^ |
| Right putamen^b^ | 0.009814^*^ | 0.01308533^*^ |
| Plasma levels of amino acids | | |
| Phenylalanine^c^ | 0.0004347^*^ | 0.0026082^*^ |
| Tyrosine^c^ | 0.002578^*^ | 0.003867^*^ |
| Leucine^c^ | 0.001862^*^ | 0.003724^*^ |
| Isoleucine^c^ | 0.003435^*^ | 0.004122^*^ |
| Valine^c^ | 0.55879 | 0.55879 |
| Phe+Tyr/BCAA ratio^c^ | 0.001439^*^ | 0.003724^*^ |

^a^ Adjustments for three cognitive domains

^b^ Adjustments for DAT availability in four regions

^c^ Adjustments for six amino acids and the related ratio

^*^ *p* value < 0.05 (two-tailed)

BCAA = branched-chain amino acid, CN = caudate nucleus, DAT = dopamine transporter, Phe = phenylalanine, Tyr = Tyrosine

**Supplementary Table S4.** The *p* values of the results of multiple regression before and after adjusting multiple comparisons

|  | *p* values (original) | *p* values (adjusted) |
| --- | --- | --- |
| DAT-cognition relationships in schizophrenia | | |
| Left CN-Attention^a^ | 0.02862^*^ | 0.02862^*^ |
| Left putamen-Attention^a^ | 0.010721^*^ | 0.021442^*^ |
| Amino acids-cognition relationships in schizophrenia | | |
| Tyrosine-Executive function^a^ | 0.0000429^*^ | 0.0000858^*^ |
| Phe+Tyr/BCAA ratio-Executive function^a^ | 0.000163^*^ | 0.000163^*^ |
| DAT-cognition relationships in all participants | | |
| Right CN-Attention^a^ | 0.7569 | 0.7569 |
| Right putamen-Attention^a^ | 0.7554 | 0.7569 |
| Left CN-Memory^b^ | 0.15340 | 0.2045333 |
| Left putamen-Memory^b^ | 0.31530 | 0.3153 |
| Right CN-Memory^b^ | 0.02531^*^ | 0.09428 |
| Right putamen-Memory^b^ | 0.04714^*^ | 0.09428 |
| Left CN-Executive function^b^ | 0.7999 | 0.8659 |
| Left putamen-Executive function^b^ | 0.8659 | 0.8659 |
| Right CN-Executive function^b^ | 0.4123 | 0.8659 |
| Right putamen-Executive function^b^ | 0.4481 | 0.8659 |
| Amino acids-cognition relationships in all participants | | |
| Phenylalanine-Attention^c^ | 0.987608 | 0.987608 |
| Tyrosine-Attention^c^ | 0.3211 | 0.80275 |
| Leucine-Attention^c^ | 0.983702 | 0.987608 |
| Isoleucine-Attention^c^ | 0.89984 | 0.987608 |
| Phe+Tyr/BCAA ratio-Attention^c^ | 0.2737 | 0.80275 |
| Phenylalanine-Memory^c^ | 0.86499 | 0.86499 |
| Tyrosine-Memory^c^ | 0.65815 | 0.86499 |
| Leucine-Memory^c^ | 0.69467 | 0.86499 |
| Isoleucine-Memory^c^ | 0.671676 | 0.86499 |
| Phe+Tyr/BCAA ratio-Memory^c^ | 0.42603 | 0.86499 |
| Phenylalanine-Executive function^d^ | 0.005295^*^ | 0.015885^*^ |
| Leucine- Executive function^d^ | 0.107776 | 0.107776 |
| Isoleucine- Executive function^d^ | 0.062272 | 0.093408 |

^a^ Adjustments for two pairs of relationships

^b^ Adjustments for four pairs of relationships

^c^ Adjustments for five pairs of relationships

^d^ Adjustments for three pairs of relationships

^*^ *p* value < 0.05 (two-tailed)

BCAA = branched-chain amino acid, CN = caudate nucleus, DAT = dopamine transporter, Phe = phenylalanine, Tyr = Tyrosine

**Supplementary References**

Yang, K.-C., Hsieh, W.-C., & Chou, Y.-H. (2022). Cognitive factor structure and measurement invariance between healthy controls and patients with major depressive disorder. *Journal of Psychiatric Research*, *151*, 598–605. doi: https://doi.org/10.1016/j.jpsychires.2022.05.029
